# The Human Pathogen *Paracoccidioides brasiliensis* Has a Unique 1-Cys Peroxiredoxin That Localizes Both Intracellularly and at the Cell Surface

**DOI:** 10.3389/fcimb.2020.00394

**Published:** 2020-08-04

**Authors:** Larissa Valle Guilhen Longo, Carlos Alexandre Breyer, Gabriela Machado Novaes, Gregory Gegembauer, Natanael Pinheiro Leitão, Carla Elizabete Octaviano, Marcos Hikari Toyama, Marcos Antonio de Oliveira, Rosana Puccia

**Affiliations:** ^1^Departamento de Microbiologia, Imunologia e Parasitologia, Escola Paulista de Medicina—Universidade Federal de São Paulo, São Paulo, Brazil; ^2^Instituto de Biociências, Universidade Estadual Paulista Júlio de Mesquita Filho, São Paulo, Brazil

**Keywords:** *Paracoccidioides brasiliensis*, 1-Cys Prx, hydroperoxides, peroxiredoxin, dimorphic fungi, ROS

## Abstract

*Paracoccidioides brasiliensis* is a temperature-dependent dimorphic fungus that causes systemic paracoccidioidomycosis, a granulomatous disease. The massive production of reactive oxygen species (ROS) by the host's cellular immune response is an essential strategy to restrain the fungal growth. Among the ROS, the hydroperoxides are very toxic antimicrobial compounds and fungal peroxidases are part of the pathogen neutralizing antioxidant arsenal against the host's defense. Among them, the peroxiredoxins are highlighted, since some estimates suggest that they are capable of decomposing most of the hydroperoxides generated in the host's mitochondria and cytosol. We presently characterized a unique *P. brasiliensis* 1-Cys peroxiredoxin (PbPrx1). Our results reveal that it can decompose hydrogen peroxide and organic hydroperoxides very efficiently. We showed that dithiolic, but not monothiolic compounds or heterologous thioredoxin reductant systems, were able to retain the enzyme activity. Structural analysis revealed that PbPrx1 has an α/β structure that is similar to the 1-Cys secondary structures described to date and that the quaternary conformation is represented by a dimer, independently of the redox state. We investigated the PbPrx1 localization using confocal microscopy, fluorescence-activated cell sorter, and immunoblot, and the results suggested that it localizes both in the cytoplasm and at the cell wall of the yeast and mycelial forms of *P. brasiliensis*, as well as in the yeast mitochondria. Our present results point to a possible role of this unique *P. brasiliensis* 1-Cys Prx1 in the fungal antioxidant defense mechanisms.

## Introduction

Paracoccidioidomycosis (PCM) is a systemic mycosis endemic in Latin America. Lethality rates range from 3 to 5% and about 80% of the PCM cases are reported in Brazilian patients (Martinez, 2017). The thermal dimorphic fungus *Paracoccidioides brasiliensis* is one of the PCM etiologic agents and the infection occurs by inhalation of fungal conidia from the environment mycelial form. The active disease, which depends on the transition to the yeast phase to occur in the lungs alveolar space, is mostly characterized by damage of the lungs and upper airways, but also of the oral mucosa and skin (Bocca et al., 2013; Martinez, 2017).

The pathogen survival depends on its mechanisms of escaping the host's immune response. An essential host defense strategy comprises the production of reactive oxygen and nitrogen species (ROS and RNS) by the immune cells, the so called respiratory burst (Campos et al., 2005; Halliwell and Gutteridge, 2015). Among the several species generated by the respiratory burst, hydrogen peroxide (H_2_O_2_), and peroxynitrite (NOO^−^) are able to generate secondary reactive species, such as organic hydroperoxides (OHPs), especially lipid hydroperoxides and other reactive species. These compounds can cause protein dysfunction and damage of biomolecules, therefore negatively affecting the pathogen homeostasis (Halliwell and Gutteridge, 2015; El-Benna et al., 2016). Several fungicides are also able to promote the ROS increase, including OHPs, which are related to fungi annihilation (Edlich and Lyr, 1992; Belenky et al., 2013; Shekhova et al., 2017). On the other hand, some fungal pathogens are able to produce OHPs to protect themselves against invasion and host tissue destruction by other microorganisms (Deighton et al., 1999).

The main enzymes involved in the pathogen antioxidant defense mechanisms against hydroperoxides are the catalase, glutathione peroxidase, and peroxiredoxins (Nevalainen, 2010). Among them, the peroxiredoxins (Prx) are noteworthy due to their high abundance in living organisms and their exceptional ability to reduce several kinds of hydroperoxides at high efficiency (10^4^-10^8^ M^−1^s^−1^) (Netto et al., 2016). In fact, some works reveal that Prxs are able to decompose most of the hydroperoxides generated in the cell (Winterbourn, 2008; Cox et al., 2009). Despite the high enzymatic efficiency, members of the Prx family differ in substrate preference between H_2_O_2_ and organic hydroperoxides (Peskin et al., 2007; Parsonage et al., 2008).

Peroxiredoxins utilize a highly reactive cysteine residue (peroxidatic cysteine—C_P_) for hydroperoxide decomposition, being classified into 1-Cys Prx and 2-Cys Prx according to the number of cysteines involved in the catalytic cycle (Rhee and Kil, 2017). The Prx catalytic triad is represented by the C_P_, a Thr/Ser, and an Arg. While C_P_ and Thr/Ser are embedded in the universal Prx motif PXXXT/SXXC_P_, the Arg residue is distant in the sequence, but it is brought closer to the Thr/Ser as a consequence of protein folding (Tairum et al., 2012, 2016).

However, specific motif signatures can be found within different classes of Prx. In the 1-Cys Prx from some fungal species, a highly conserved PV/TC_P_TTE motif is found (Nevalainen, 2010; Rocha et al., 2018). The most studied member of the 1-Cys Prx is the mammalian Prdx6, which is a dual-function enzyme with both peroxidase and acidic Ca^2+^-independent phospholipase A2 activities (Nevalainen, 2010; Zhou et al., 2018). This additional function seems to protect cell membrane phospholipids against oxidative damage (peroxidation) and hydrolysis. Remarkably, some 1-Cys Prx from pathogens have distinguished features in comparison with the corresponding host enzymes, placing them as promising targets for the development of specific drugs (Wen et al., 2007; Rocha et al., 2018).

Previous work from our group revealed the presence of an ortholog of a mitochondrial peroxiredoxin (PbPrx1) in the cell wall proteome from both the yeast and the mycelial forms of *P. brasiliensis* (Longo et al., 2014). Importantly, this protein has also been described in the proteome of extracellular vesicle (EV) of this and other human fungal pathogens (Vallejo et al., 2012). More recently, we have observed by quantitative proteome that PbPrx1 is 2–3-fold more abundant in EVs isolated from *P. brasiliensis* cell supernatants after nitrosative and oxidative stress (Leitão et al., unpublished).

Considering the possible role of peroxiredoxins in the fungal defense mechanisms against the host immune system, we presently aimed at characterizing PbPrx1. We initially found that it corresponds to a unique 1-Cys Prx sequence in the *P. brasiliensis* genome. The corresponding protein was recombinantly expressed, purified, and the enzymatic activity was characterized. Our results point to a possible role of this unique *P. brasiliensis* 1-Cys Prx1 in the fungal antioxidant defense mechanisms.

## Materials and Methods

### Fungal Strains and Culture Conditions

*P. brasiliensis* (isolate Pb18) was cultivated as previously described (Vallejo et al., 2011). Overall, yeast cells were maintained in modified YPD (0.5% yeast extract, 0.5% casein peptone, 1.5% glucose, pH 6.5) slants at 4°C. For the experiments, yeasts were recovered by seeding into fresh slants for growth for 7 days at 36°C. Actively growing yeasts were inoculated into liquid Ham's F12 medium (Life Technologies, Grand Island, NY, USA) supplemented with 1.5% glucose (F12/glc) for pre-growth under shaking for 4 days at 36°C. The cells were then transferred to fresh F12/glc and cultivated for 2 extra days. Viability (>95%) was estimated by trypan blue staining.

### RNA Extraction and cDNA Cloning

For RNA extraction, freshly grown cells suspended in TRIzol reagent (Thermo Fisher Scientific, Waltham, MA, USA) were mechanically disrupted by vortexing with glass beads for 10 min or submitted to a Precellys 24 high-throughput homogenizer (Bertin Technologies, Rockville, Washington, DC, USA). Total RNA was then isolated following chloroform extraction and isopropanol precipitation. Genomic DNA was removed with RNase-free DNase I (Promega Corp., Madison, WI, USA), as previously described (Goldman et al., 2003). The efficiency of hydrolysis was tested by PCR amplification of the Pb*GP43* gene using primers that included (or not) the introns (Cisalpino et al., 1996). Three micrograms of total RNA were reverse transcribed using SuperScript III reverse transcriptase (Thermo Fisher Scientific, Waltham, MA, USA) and oligo(dT)_12−18_ primers. Using the cDNA as template, a 680-bp fragment was amplified by PCR using the PbPrx1-F and PbPrx1-R primers (Table 1) and Taq Platinum High Fidelity enzyme. The Pb*PRX1* amplified fragment was cloned into pGEM-T easy (Promega Corp., Madison, WI, USA) at the *Bam* HI/*Hind* III sites and a recombinant plasmid was selected in *Escherichia coli* DH5α resistant to the ampicillin marker. Correct in-frame ligation was checked by endonuclease restriction and sequencing. The insert was excised from pGEM-T easy using *Bam* HI/*Hind* III (New England Biolabs, Ipswich, MA, USA) and subcloned into the same sites of both the pHIS1 (pHIS1*-pbPRX1*) or the pET28PP (pET28PP*-pbPRX1*) expression vectors (Novagen, WI, USA). The resulting plasmids were used for expression of the recombinant protein, respectively in *E. coli* BL21(DE3) pLys-S (Novagen, Madison, WI, USA), to be used in antibody production, or *E. coli* BL21 Tuner (DE3) (Novagen, Madison, WI, USA), for biochemical analysis. The recombinant protein pHIS1*-pbPRX1* was not soluble and yielded only 2 mg/ml, but that was enough to immunize mice for antibody production. The recombinant protein pET28PP*-pbPRX1*, on the other hand, was produced in a later phase of the work specifically to perform complete biochemical analysis, which demands high amounts of enzyme. It was soluble and yielded 20 mg/mL.

**Table 1:** Primers used in PCR and sequencing during this study.

**Name**	**Application**	**Sequence**
Prx1-F	Cloning	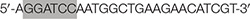
Prx1-R	Cloning	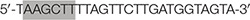
Gp43-F	DNAse control	5′-GGGACACCTTTATCACT-3′
Gp43-R	DNAse control	5′-CCAAGACATACAAGAACGTC-3′
T7_pHIS1_	Sequencing	5′-TAATACCACTCACTATAGGG-3′
T7 terminator	Sequencing	5′-GCTAGTTATTGCTCAGCGG-3′
T7_pGEM_	Sequencing	5′-TAATACGACTCACTATAGGG-3′
SP6	Sequencing	5′-ATTTAGGTGACACTATAGAA-3′
Prx1-F	qPCR	5′-ATTTCACTCCTACCTGCAC-3′
Prx1-R	qPCR	5′-CGTTGATGTCGTTGATCCAG-3′
TUB-1	qPCR	5′-CGGCTAATGGAAAATACATGGC-3′
TUB-2	qPCR	5′-GTCTTGGCCTTGAGAGATGCAA-3′

*BamHI and HindIII restriction sites are highlighted in gray*.

### Protein Expression and Purification for Biochemical Analysis

Single colonies of *E. coli* BL21 Tuner (DE3) harboring the pET28PP*-pbPRX1* vector were cultured overnight at 37°C in 2XYT medium containing ampicillin (100 μg mL^−1^), diluted in fresh medium and grown to an *OD*_600_ = 0.6–0.8. IPTG (0.5 mM final concentration) was added and the cells were incubated for 16 h at 30°C, at 250 rpm. The cells were harvested by centrifugation and the pellet was washed, suspended in start buffer (10 mM Tris-Cl pH 8.0, 1% Triton), and sonicated (three cycles of 30 s, 30% amplitude, 60 s in ice). Cell extracts were treated for 15 min with 1% streptomycin sulfate in ice and the suspensions were centrifuged (12,000 rpm, 60 min) to remove nucleic acid precipitates and insoluble components. Cell extracts were purified by IMAC using His-Trap columns (GE Healthcare, Piscataway, USA) eluted with an imidazole gradient. Imidazole was removed by gel filtration using a PD10 column (GE Healthcare, Piscataway, USA), the His-tag was excised using HRV3C protease (Novagen, Madison, WI, USA) and the reaction was performed overnight in 10 mM Tris-Cl (pH 8.0). His-Tag and HRV3C protease were separated from recombinant rPbPrx1 by IMAC (Supplementary Figure 1A). The thioredoxin 1 (Trx1), thioredoxin reductase 1 (TrxR1), and thiol specific antioxidant 1 (Tsa1) from *Saccharomyces cerevisiae* were expressed and purified as previously described (Oliveira et al., 2010). Briefly, *E. coli* BL21 (DE3) cultures containing the pET15b/*tsa1*, the pPROEX/*trxr1*, and the pET17/*trx1* vectors were stimulated with 1 mM IPTG for 3 h. The cells were lysed and centrifuged to remove the precipitate. The Tsa1 and TrxR1 recombinant proteins (Supplementary Figures 1B,C) were purified by imidazole gradient (0–0.5 M imidazole) using a Hi-Trap column (GE Healthcare, Piscataway, USA). Trx1 purification was carried out by size exclusion chromatography (Supplementary Figure 1D) using a HiLoad 16/600 Superdex 75 column (GE Healthcare, Piscataway, USA). The enzyme concentration was determined spectrophotometrically by molar extinction coefficient (rPbPrx1 ε_280_ = 22,920 M^−1^ cm^−1^; Tsa1ε280 = 23,950; Trx1ε_280_ = 9,970 M^−1^ cm^−1^, and TrxR1 ε_280_ = 24,410 M^−1^ cm^−1^). The recombinant protein samples were stored at −20°C.

#### Mice Immunization

Recombinant PbPrx1 (rPbPrx1) expressed from the pHIS1*-pbPRX1* vector was purified in Ni-NTA agarose (Qiagen, Germantown, MD, USA). Purified rPbPrx1 (100 μg) was emulsified in incomplete Freund's adjuvant and inoculated subcutaneously in Balb/C mice at days 0, 15, and 30, when the sera were collected, tested, and aliquoted at −20°C. Control pre immune serum was obtained before immunization. Monospecific affinity-purified pre-immune and immune anti-rPbPrx1 mouse sera were prepared as previously described (Batista et al., 2006). Briefly, the purified rPbPrx1 was immobilized onto nitrocellulose membranes and incubated with pre-immune and immune anti-rPbPrx1 mouse sera. The affinity-purified antibodies were eluted and stored at −20°C until use. The use of animals in this work was reviewed and approved by the Ethics Committee for Research (UNIFESP) numbers CEP 0366/07 and CEP 6379211014.

### Confocal Microscopy and Immunoblotting

For confocal microscopy, fungal cells were fixed in 4% paraformaldehyde for 20 min at room temperature, washed twice in PBS and permeabilized in Triton X-100 0.5% for 15 min. Fixed cells were washed three times in PBS and quenched with 3% bovine serum albumin (Sigma-Aldrich, St. Louis, MO, USA) in PBS (blocking buffer) for 16 h at 4°C. Quenched cells were incubated with primary antibodies at 1:50 (monospecific pre-immune and anti-rPbPrx1 mouse serum) in blocking buffer for 2 h at 37°C, washed three times in PBS and incubated for 1 h at 37°C in the dark with secondary anti-mouse-IgG (1:100) labeled with both Alexa (Alexa FluorR 488, Invitrogen) and 25 μM Calcofluor White. Microscopy slides were mounted with anti-fading Vectashield (Vector Laboratories, Burlingame, CA, USA) and sealed. Images were analyzed by confocal microscopy (Carl Zeiss LSM-510 NLO, Oberkochen, BadenWurttemberg, Germany).

Western immunoblot reactions were performed with immune and pre immune anti-rPbPrx1 sera against cell wall and mitochondrial extracts. Incubation was carried out overnight at 4°C under shaking. The membranes were washed three times in 0.1% Tween 20 diluted in PBS and incubated for 1 h at 37°C with goat anti-rabbit conjugated to peroxidase (Sigma). The reactions were developed using an enhanced chemiluminescence kit (ECL reagent, Pierce, Rockford, IL, USA). Cell wall and mitochondrial extracts were produced as described elsewhere (Batista et al., 2006; Longo et al., 2014). Briefly, for extraction of cell wall proteins*, P. brasiliensis* yeast and mycelial cells were washed three times in ice-cold 25 mM Tris–HCl, pH 8.5, and incubated with 2 mM DTT in the same buffer supplemented with 1 mM phenylmethylsulfonyl fluoride (PMSF) and 5 mM ethylenediaminetetraacetic acid (EDTA) to inhibit the action of proteases. For isolation of mitochondria, nitrogen-frozen yeast cells were mechanically disrupted in a mortar, thawed in 0.6 M sorbitol, 20 mM Hepes, pH 7.4, and sonicated for 5 min. Cell debris were pelleted (1,500 × g, 5 min) and the mitochondrial fraction was precipitated from the supernatant (1,200 × g, 10 min).

### Flow Cytometry Analysis

For fluorescence-activated cell sorter (FACS) analysis, *P. brasiliensis* yeast cells were prepared as previously described (Soares et al., 1998), with modifications. Fungal cells were fixed for 1 h at room temperature in 4% (vol/vol) paraformaldehyde in PBS, pH 7.2. Fixed cells were precipitated by centrifugation (1 min at 5,600 × g), washed three times in PBS and quenched for 1 h at room temperature in PBS containing 1% BSA (blocking buffer). The cells were incubated overnight at 4°C with monospecific anti-rPbPrx1 or pre-immune control (1:100 in blocking buffer). After five washes in PBS, the cells were incubated in the dark for 1 h at 37°C with Alexa-488-anti-rabbit IgG (Sigma-Aldrich) at 1:300 in blocking buffer. Labeled cells were rinsed five times, resuspended in PBS, and analyzed in a FACS Canto II (BD Biosciences, San Jose, CA, USA). A total of 10,000 cells were analyzed for fluorescence at 492 to 520 nm. Unlabeled control cells were previously analyzed for autofluorescence, relative cell size, and granularity.

### qPCR Analysis

For evaluation of the Pb*PRX1* expression in *P. brasiliensis* undergoing oxidative stress, logarithmic yeast cells growing in F12/glc were subdivided into aliquots and centrifuged. Cell pellets were resuspended in 5 mL PBS containing or not (control) 5 mM H_2_O_2_, cumene hydroperoxide (CHP), or tert-butyl peroxide (*t*-BOOH), and incubated for 15, 30, and 60 min. Cell pellets were collected by centrifugation, washed in PBS, frozen in liquid N_2_ and kept at −80°C until RNA extraction. Quantitative PCR (qPCR) was performed in triplicate using a SYBR-green-based PCR master mix (Applied Biosystems, Foster City, CA, USA) with reverse-transcribed RNA template and 0.5 M of each primer (Table 1). Cycling was carried out in triplicate in a Real-Time 7500 thermocycler (StepOnePlus™ Real-Time PCR System—Applied Biosystems™) starting with a holding stage at 95°C (10 min), followed by 40 cycles at 95°C (15 s) and 60°C (60 s). The dissociation curve was determined with an additional cycle of 95°C (15 s), 60°C (60 s), and 95°C (15 s). Changes in the transcript levels were determined using the threshold cycle (ΔΔCT) method (Schmittgen and Livak, 2008) after normalization of cycle thresholds based on the expression of the alpha-tubulin gene (XM_010765319.1). The alpha-tubulin gene is a standard normalizing gene for qPCR in *P. brasiliensis*, considering that its expression is stable and does not tend to fluctuate even under oxidative stress (Grossklaus et al., 2013). Statistical significance was determined by the Student's *t*-test.

### Characterization of the rPbPrx1 Thiol-Dependent Peroxidase Activity by the DTT Oxidation Assay

rPbPrx1 (12.5 μM) expressed in pET28PP—*pbPRX1* was incubated for 10 min in a solution containing 10 mM 1,4-dithiothreitol (DTT), 5 mM H_2_O_2_ or *t*-BOOH, 100 μM diethylenetriaminepentaacetic acid (DTPA), and 1 mM sodium azide in 10 mM Hepes-NaOH, pH 7.4. The rate of DTT oxidation was measured spectrophotometrically at 310 nm (ε_280_ = 110 M^−1^ cm^−1^) at 30°C, as previously described (Tairum et al., 2012).

### rPbPrx1 Peroxidase Inactivation by NEM Alkylation

To determine whether the peroxidase activity is cysteine-dependent, rPbPrx1 (2 mg mL^−1^) was treated with DTT for 1 h at room temperature. The excess DTT was removed by gel filtration using a PD-10 desalting column (GE Healthcare, Piscataway, USA). The reduced protein was incubated in 1 mM N-ethylmaleimide (NEM) (Sigma, München, Germany) overnight at 4°C. The excess NEM was removed by gel filtration using a PD-10 desalting column (GE Healthcare, Piscataway, USA). N-Ethylmaleimide (NEM) is an alkylating reagent that reacts with sulfhydryl groups, thus blocking the Prx activity. The reactions were performed at 30°C in a solution containing 50 mM Hepes-NaOH (pH 7.4), 100 μM DTPA, 1 mM sodium azide, 12.5 μM rPbPrx1, 10 mM DTT, and 5 mM *t*-BOOH. The peroxidase activity was monitored by the DTT oxidation (λ = 310 nm).

### Ferrous Oxidation Xylenol Orange (FOX) Assay

rPbPrx1 hydroperoxide activity was determined using the FOX assay (Nelson and Parsonage, 2011). Reactions were prepared to a final volume of 50 μL in 50 mM Hepes-NaOH (pH 7.4), 5 μM rPbPrx1, 100 μM sodium azide, 100 μM DTPA, 1 mM DTT, and 150 μM hydroperoxide [H_2_O_2_, *t*-BOOH, CHP, and linoleic acid hydroperoxide (L-OOH)] and incubated at room temperature. To investigate additional electron donors of rPbPrx1, decomposition of *t*-BOOH was monitored using 1 mM DTT, 3 mM GSH, 3 mM β-mercaptoethanol, or 300 μM DHLA as reducing agents.

### Thioredoxin-Dependent Peroxidase Activity Using the *S. cerevisiae* Trx System

Thioredoxin peroxidase activity was evaluated using the heterologous cytosolic thioredoxin system from *S. cerevisiae* by monitoring the NADPH oxidation. The reaction was carried out at 30°C in a final 100 μL volume containing 50 mM Hepes-NaOH, pH 7.4, 150 μM NADPH, either 200 μM *t*-BOOH or H_2_O_2_, 1 μM rPbPrx1, 1 μM *S. cerevisiae* Trx1, and 0.3 μM *S. cerevisiae* TrxR1. Positive control was performed using 1 μM Tsa1. The reaction was initiated by the addition of hydroperoxide and NADPH oxidation was monitored at 340 nm (ε_340_ = 6220 M^−1^ cm^−1^). Negative control was performed without the addition of peroxidases.

### Evaluation of the rPbPrx1 Phospholipase Activity

The rPbPrx1 phospholipase activity was evaluated using an adaptation of the method described by Petrovic et al. (2001). Reaction mixtures (250 μL) containing 40 μM rPbPrx1 in 10 mM Tris-Cl pH 8.0 were started with 25 μL 4-nitro-3-octanoyloxy benzoic acid (NOBA) solubilized in acetonitrile 100% to a final concentration of 3 mg mL^−1^. Reactions were maintained at 25°C for 60 min and absorbance (*A*_425nm_) was recorded. The *Rattus norvegicus* Prx6 was used as a positive control of the phospholipase activity.

### Determination of Thermal Stability

Thermal shift assays for rPbPrx1 were performed by circular dichroism (CD). CD spectra of rPbPrx1 were obtained using a 0.1-cm path length cuvette containing 5 μM of protein sample in 10 mM Tris buffer, pH 8.0. Assays were carried out in a Jasco J-810 spectropolarimeter (Jasco Inc., Tokyo, Japan) at temperatures varying from 20 to 80°C, at an increment of 2°C min^−1^. Melting temperatures (*T*_m_) were calculated by fitting the sigmoidal melting curve to the Boltzmann equation using GraphPad Prism version 5.01 (GraphPad Prism Software, San Diego, USA), with R2 values of >0.98. The spectra are shown as an average of eight scans recorded from 190 to 260 nm. The content of secondary structures was estimated using the CDNN 2.1 software (Bohm et al., 1992).

### Analysis of the rPbPrx1 Quaternary Structure by Size-Exclusion Chromatography

Size-exclusion chromatography experiments were performed by analytical HPLC equipped with a PU 2880 Plus injector and a PDA MD 2018 detector (LC-2000 series; Jasco, Tokyo, Japan). The samples (50 μM in 100 mM Tris-HCl, pH 7.4) were separated by a system containing a Phenomenex BioSep-SEC-S3000 column (7.8 × 300 mm, 5 μm, resolution range of 1–300 kDa, Phenomenex, Inc., Torrance, California, USA) at a flow rate of 1.0 mL min^−1^ in 100 mM Tris-HCl, pH 7.4, containing 50 mM NaCl. The elution profile was monitored by absorbance at λ = 280 nm. Bovine thyroglobulin (670 kDa), bovine gamma globulin (158 kDa), ovalbumin (44 kDa), myoglobin (17 kDa), and vitamin B_12_ (1.35 kDa) were used as molecular standards (Bio-Rad Laboratories, Richmond, USA). Chromatograms were analyzed using Jasco BORWIN, version 1.50, software (Jasco, Tokyo, Japan). The redox treatments for rPbPrx1 were either 5 mM TCEP (reductant) or 1.2 molar equivalent of hydrogen peroxide (oxidant) for 30 min, at 25°C prior to the chromatographic runs.

## Results

### The *P. brasiliensis* Genome Bears a Single 1-Cys Prx-like Gene

Database search (https://www.ncbi.nlm.nih.gov) showed that *P. brasiliensis* has a single Prx1 ortholog (PbPrx1) that is 222-amino-acid long and has a deduced molecular mass of 24.7 kDa. Amino acid sequence alignments of PbPrx1 with 1-Cys Prx1 from other species shows that PbPrx1 has only one conserved cysteine (Cys51; Supplementary Figure 2, red asterisk), which corresponds to the Cys91 from *S. cerevisiae* ScPrx1. The signature sequence PVC_P_TTE, characteristic to the 1-Cys Prx1 group, carries a point substitution of the second residue (V → T) resulting in the PTC_P_TTE sequence, which is also observed in other phylogenetically-related temperature-dependent dimorphic species such as *P. lutzii, H. capsulatum*, and *Blastomyces dermatitidis* (Supplementary Figure 2). Overall, the PbPrx1 sequence showed 57% identity and 72% similarity to the 1-Cys Prx1 isoform from ScPrx1. As expected, we observed higher homology with the isoforms from *H. capsulatum* (HcPrx1; 89% identity) and *B. dermatitidis* (BdPrx1; 87% identity), while the identity between PbPrx1 and PlPrx1 is 98%. In Supplementary Figure 2, the green box shows a Ca^2+^-independent phospholipase A2 (PLA2) motif GDSWG (Nevalainen, 2010) that is not conserved in PbPrx1, PlPrx1, HcPrx1, or BdPrx1. In these sequences, the Ser residue involved in catalysis is substituted for Lys/His (GDK/HYV). Importantly, PbPrx1 does not have an *N*-terminal mitochondrial signal peptide, which can be seen in the Prx1 isoforms from *S. cerevisiae, Aspergillus nidulans*, and *Candida albicans*, thus suggesting a cytosolic localization of the protein (Supplementary Figure 2).

### PbPrx1 Cellular Location

**Figure 1:**
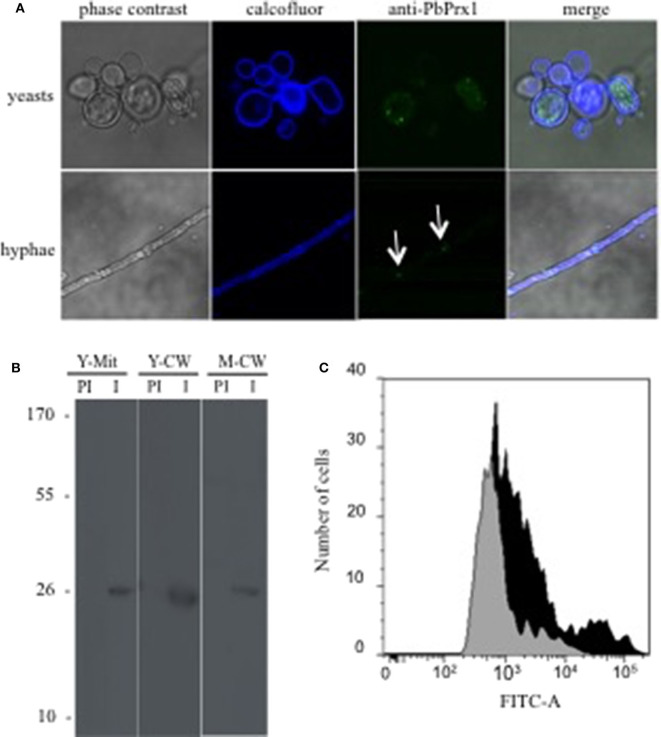
*P. brasiliensis* PbPrx1 localizes both in the cytoplasm and at the cell wall. **(A)** Confocal microscopy of yeast and mycelial cells. In blue, Calcofluor White staining of the cell wall. In green, reactivity with anti-rPbPrx1 monospecific antibodies (1:50). **(B)** Western blot reaction of cell wall (CW) and mitochondrial (Mit) extracts from the yeast (Y) and mycelial (M) fungal phases with anti-rPbPrx1 (I) and preimmune monospecific antibodies (PI, 1:100). The molecular mass is indicated on the left in kilodaltons (kDa). **(C)** FACS analysis of *P. brasiliensis* non-permeabilized yeast cells incubated with either anti-rPbPrx1 (black) or preimmune (gray) antibodies (1:100).

In order to study the PbPrx1 cell localization, we produced mice anti-rPbPrx1 immune sera, using a recombinant rPbPrx1 protein as immunogen, and monospecific anti-PbPrx1 antibodies. Confocal microscopy images seen in Figure 1A show fluorescence label in the cytoplasm, in a punctuated pattern, in both the yeast and mycelial phases of *P. brasiliensis*. Interestingly, PbPrx1 seems to accumulate close to hyphal septa (white arrows). Cell wall and mitochondrial localization were further investigated by immunoblot (Figure 1B), which revealed a 25-kDa protein band reacting with anti-rPbPrx1 antibodies in the yeast mitochondrial extracts, even though the PbPrx1 sequence lacks an *N*-terminal mitochondrial signal peptide (Supplementary Figure 2). Similarly, anti-rPbPrx1 antibodies specifically reacted with a single protein band of approximately 25 kDa in cell wall extracts from *P. brasiliensis* yeasts and mycelia, suggesting that PbPrx1 colocalizes at the *P. brasiliensis* yeast cell wall. Although cell wall localization was not clear in confocal images, PbPrx1 surface labeling has also been suggested by FACS analysis of non-permeabilized *P. brasiliensis* yeast cells labeled with anti-rPbPrx1 antibodies (Figure 1C). Anti-rPbPrx1 immune serum reacted with yeast cells with higher fluorescence intensity than pre-immune serum (5-fold increase), suggesting that the reaction was specific. The number of positive cells was also 50% higher in the reaction with anti-rPbPrx1 immune serum than with pre-immune serum. Taken together, our results suggest that PbPrx1 localizes to the cytoplasm and cell wall of the yeast and mycelial forms of *P. brasiliensis*, as well as in the yeast mitochondria.

### Biochemical Characterization of rPbPrx1

**Figure 2:**
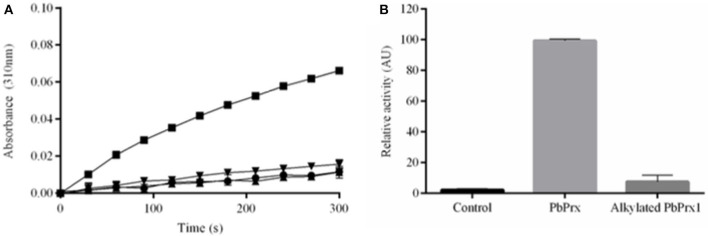
The rPbPrx1 peroxidase activity is thiol-dependent. **(A)** The rPbPrx1 peroxidase activity was monitored by the DTT oxidation assay (λ = 310 nm). The reactions were performed with 12.5 μM rPbPrx1 at 30°C with either 5 mM H_2_O_2_ (▾) or *t*-BOOH (■). Control reactions were performed in triplicate, at least three times, with either *t*-BOOH (•) or H_2_O_2_ (▴) in the absence of enzyme. **(B)** To determine the peroxidase cysteine-dependent activity, rPbPrx1 was reduced by treatment with DTT for 1 h at room temperature. The reactions were performed as described above for (■). The peroxidase activity was monitored by the DTT oxidation assay (λ = 310 nm) and the results are shown as relative activity in graphic bars. All experiments were performed at least three times and yielded similar results.

To determine whether *P. brasiliensis* PbPrx1 is a thiol-dependent peroxidase, the activity for both H_2_O_2_ and *t*-BOOH consumption was monitored by DTT oxidation. As indicated in Figure 2, both hydroperoxides were reduced, although the enzyme exhibited higher affinity for *t*-BOOH than for H_2_O_2_ (*v*_0_ = 0.89 and 2.73 μM s^−1^, respectively). Peroxidatic cysteine-dependent activity was confirmed by NEM alkylation, corroborating with the evidence that the PbPrx1 is a peroxiredoxin (Figure 2B). To evaluate the rPbPrx1 phospholipase activity, we performed a phospholipase assay using 4-Nitro-3-(octanoyloxy) benzoic acid (NOBA). As predicted by structural analysis, the PbPrx1 does not have phospholipase activity (Supplementary Figure 2). Together, our results show that PbPrx1 is a thiol-dependent peroxidase that has higher affinity for organic hydroperoxides, but that lacks phospholipase activity.

**Figure 3:**
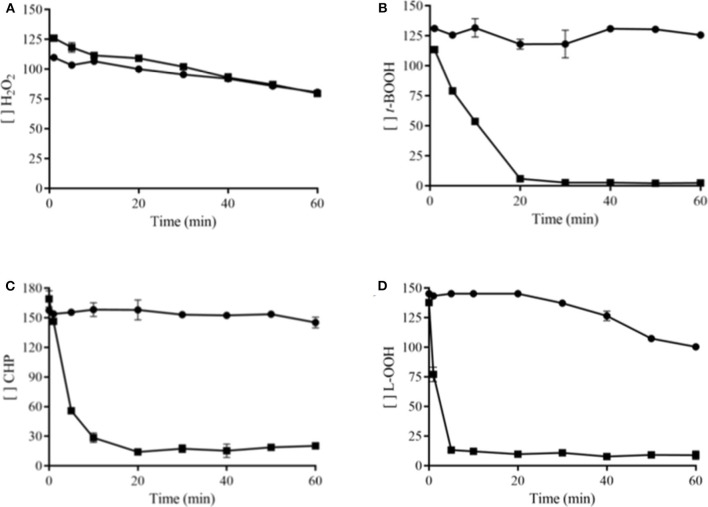
rPbPrx1 and hydroperoxide specificity. The FOX assay was performed at room temperature with 5 μM rPbPrx1 (■) or without protein (•, negative control), using 150 μM of H_2_O_2_ **(A)**, *t*-BOOH **(B)**, CHP **(C)**, or L-OOH **(D)** as substrate. The absorbance was monitored at 560 nm. The graphics were generated using GraphPad Prism and show the remaining concentration of hydroperoxides during a 60-min reaction time. All experiments were performed in triplicate and repeated at least three times yielding consistent results.

In order to confirm that organic substrates are decomposed more efficiently by PbPrx1, we also performed a FOX assay with both H_2_O_2_ and different organic hydroperoxides such as tert-butyl (*t*-BOOH), cumene (CHP), and linoleic hydroperoxides (L-OOH). The enzyme catalytic efficiency was significantly higher for L-OOH (Figure 3D), with initial velocity (*V*_0_) of 12.12 ± 1.2 μM min^−1^, which is ~2.7-fold higher than that for CHP (4.52 ± 0.06 μM min^−1^) and ~8.8-fold higher than that stimated for *t*-BOOH (1.37 ± 0.02 μM min^−1^) (Figures 3B–D). On the other hand, the enzyme did not show significant decomposition rate for hydrogen peroxide (0.024 μM min^−1^) (Figure 3A). Thus, our results confirm that rPbPrx1 has pronounced affinity for organic peroxides, particularly for more hydrophobic OHPs (CHP and LOOH). That may indicate the presence of a hydrophobic microenvironment in the active site of the enzyme, as observed for some Prx (Hall et al., 2009, 2011).

### Pb*PRX1* Gene Expression

**Figure 4:**
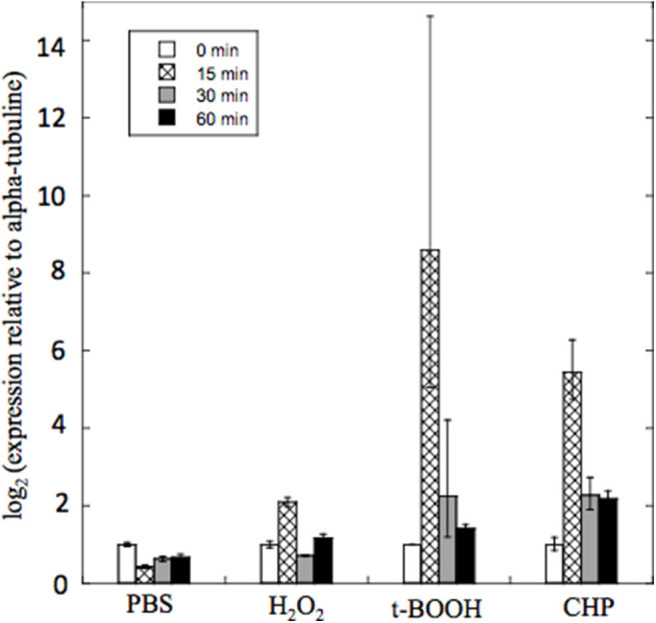
Quantitative real time RT-PCR of Pb*PRX1* from Pb18 yeast cells after oxidative stress. The cells were incubated for 60 min in PBS containing 5 mM H_2_O_2_, *t*-BOOH, or CHP (cumene hydroperoxide). Changes in transcript levels were determined in comparison to incubation with PBS alone. The cycle thresholds were normalized with the expression of the alpha-tubulin gene.

To gain insight into the physiological roles of PbPrx1 during oxidative stress, its expression pattern was characterized when yeast cells were exposed to both inorganic (H_2_O_2_) and organic (*t*-BOOH and CHP) peroxides for 15, 30, and 60 min (Figure 4). As a general trend, the Pb*PRX1* gene was regulated in a time-dependent manner, with higher expression levels at 15 min, followed by decreased expression in the following timepoints. Pb*PRX1* gene expression was induced by the three hydroperoxides, but a more pronounced upregulation was seen with organic peroxides. Pb*PRX1* gene expression increased 8.5- and 5.4-fold, respectively for *t*-BOOH and CHP, in comparison to only 2-fold for H_2_O_2_. These results infer that PbPrx1 might have a more important physiological role during oxidative stress caused by organic hydroperoxides, which is consistent with the higher affinity of PbPrx1 for these molecules (Figure 3).

### Analysis of rPbPrx1 Reductants

**Figure 5:**
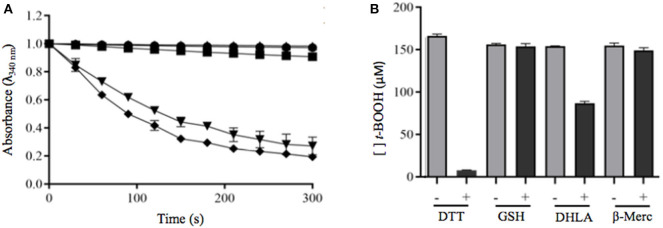
Evaluation of dithiolic and monothiolic compounds as electron donors to rPbPrx1. **(A)** Trx-linked rPbPrx1 peroxidase activity was evaluated using the *S. cerevisiae* heterologous Trx system and rPbPrx1 by monitoring the NADPH oxidation (λ = 340 nm) at 30°C in the presence of either *t*-BOOH (•) or H_2_O_2_ (■). Reactions without rPbPrx1 were used as negative controls (▴). As positive controls, we used samples containing *S. cerevisiae* Tsa1 (*t*-BOOH, ▾; H_2_O_2_, ♦). **(B)** FOX assay evaluation of rPbPrx1 thiol-dependent peroxidase activity using different reductant thiolic compounds. The peroxidase activity was evaluated from the amount of remaining hydroperoxide (λ = 560 nm). The reactions were performed using different reducing agents: GSH (3 mM), β-mercaptoetanol (3 mM), and DHLA (300 μM). The bars represent the final concentration of *t*-BOOH after 15 min. All experiments were performed at least three times and yielded similar results.

To investigate the possible PbPrx1 reductants, the following reducing agents were tested: DTT and DHLA (dithiolic), GSH and β-ME (monothiolic), and the heterologous *S. cerevisiae* Trx system, which is responsible for Prx reduction in this organism. It can be inferred from the results seen in Figure 5B that rPbPrx1 only receives electrons from dithiolic compounds of low molecular weight (DTT and DHLA), but not from monothiolic compounds (GSH and β-ME) or from the heterologous *S. cerevisiae* Txr reducing system compounds (Figure 5A), although they have been described as possible reducing agents to other 1-Cys Prx (Pedrajas et al., 2000; Monteiro et al., 2007; Rocha et al., 2018).

### Evaluation of the rPbPrx1 Secondary and Quaternary Structure

**Figure 6:**
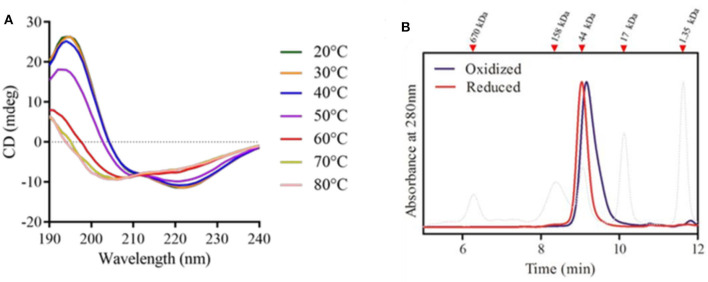
rPbPrx1 structural analysis by circular dichroism (CD). **(A)** rPbPrx1 thermal shift assay. rPBPrx1 (5 μM) was evaluated at different temperatures (20–80°C) in 10 mM Tris buffer (pH 8.0) and the results are presented as an average of eight scans recorded from 190 to 260 nm. All spectra were corrected against the buffer. The table showing the percentage of secondary structures in accordance with the temperature profile is available as Supplementary Table 1. **(B)** Size-exclusion chromatography of rPpPrx1 in reduced (red line) and oxidized (blue line) forms was performed in a BioSep-SEC-S2000 column and monitored by absorbance at 280 nm. The elution profile of molecular standards is represented by dotted lines. All experiments were performed at least three times with similar results.

Recombinant rPbPrx1 samples were analyzed by CD to assess the structural content and conformational changes related to temperature. Our results indicate the presence of an α/β protein with secondary structure composed of ~36% α-helices, ~27% β structures, and ~37% unstructured regions (Figure 6A, Supplementary Table 1), similar to what has been observed for 1-Cys Prx from other organisms (Choi et al., 1998; Sarma et al., 2005). The enzyme has poor thermal stability, maintaining its native conformation only at temperatures up to 40°C (Figure 6A), which, on the other hand, is compatible with its activity in the human host.

Size exclusion chromatography was performed to analyze rPbPrx1 quaternary structure. The elution pattern shows single peaks compatible with molecules of ~45 kDa, suggesting that the enzyme is mainly found in a dimeric form. rPbPrx1 analysis in different redox states indicates that the oxidized structure elutes more compactly than that observed in the reduced state (Figure 6B), however maintaining the dimeric structure.

## Discussion

In the present work, we characterized a single Prx1 ortholog found in the genome of the dimorphic fungus *P. brasiliensis* (PbPrx1). The PbPrx1 protein analysis showed that the universal motif PVC_P_TTE of the 1-Cys Prx sequences contains a substitution of the second aminoacidic residue (Val), a hydrophobic residue, to a polar Thr (PTC_P_TTE). This substitution is shared by other dimorphic fungi, which may suggest a characteristic signature for this group of fungal species. In another related group of fungal species, specifically, *A. niger, A. oryzae*, and *C. tropicalis*, the Val residue is substituted by Ile (PIC_P_TTE). Since this amino acid is in the vicinity to the catalytic cysteine (C_P_), the mentioned substitutions may affect both the catalytic activity of the Prx and its substrate preference. On the other hand, the sequence analysis revealed the inexistence of a phospholipase C-terminal motif, which is in agreement with our experimental results indicating that PbPrx1 does not show phospholipase activity. So far, there are only two fungal 1-Cys Prx1 bearing phospholipase activity, namely the AfPrx1 and AfPrxC isoforms from *A. fumigatus* (Bannitz-Fernandes et al., 2019). Concerning the secondary and quaternary structures, the PbPrx1 does not differ significantly from that of other organisms in the secondary structure content (Gretes et al., 2012). As to the PbPrx1 quaternary structure, we found a dimer, while both dimers and monomers have been observed in the human Prx6 by SEC (Wu et al., 2006).

We also demonstrated that the thiol peroxidase activity is dependent on a Cys residue and that the enzyme has higher affinity for *t*-BOOH than for H_2_O_2_. The fact that PbPrx1 can decompose organic hydroperoxides more efficiently than hydrogen peroxide is not an exclusive characteristic of PbPrx1. Other thiol peroxidases, such as the human PrxV, *E. coli* thiol peroxidase (EcTpx), and the organic hydroperoxide resistance protein (Ohr) from *Xylella fastidiosa* are 100–1000-fold more reactive with organic peroxides, as a consequence of a hydrophobic active site microenvironment, which enables interactions with hydrophobic oxidizing substrates (Cussiol et al., 2003; Perkins et al., 2014; Alegria et al., 2017; Piccirillo et al., 2018). In this context, our data suggest that the active site microenvironment of PbPrx1 is also highly hydrophobic, suggesting that PbPrx1 may act as a strong scavenger of organic peroxides in *P. brasiliensis* cells. Regarding the reductant substrates, we showed that PbPrx1 is not able to receive electrons from a heterologous *S. cerevisiae* thioredoxin reducing system or from monothiolic compounds (GSH and β-ME). The recombinant PbPrx1 was only reduced by dithiolic compounds of low molecular weight (DTT and DHLA).

We have previously found the PbPrx1 protein in the cell wall proteome from both the yeast and mycelial forms of *P. brasiliensis*, isolates Pb18 and Pb3, and in the extracellular vesicle (EV) proteome from *P. brasiliensis* Pb18 (Longo et al., 2014). In Pb18, PbPrx1 was 2–3-fold more abundant upon nitrosative and oxidative stress (Leitão et al., unpublished), suggesting a role for the enzyme during stress. We have presently shown predominant cytoplasmic localization of PbPrx1 in *P. brasiliensis* by confocal microscopy, but the protein was additionally labeled in cell wall extracts and in the surface of non-permeabilized yeast cells by FACS analysis. Surprisingly, the PbPrx1 was also identified in mitochondrial protein extracts from yeast cells by Western blot, despite the fact that the predicted sequence lacks an *N*-terminal mitochondrial signal peptide. Although we cannot discard that the mitochondrial extract may be contaminated with small amounts of cytoplasmic PbPrx1, it is more likely to assume that PbPrx1 is not directed to the mitochondria through the classical import pathway guided by amino-terminal presequences, but instead through internal targeting signals, as seen for more than 50% of mitochondrial proteins (Bolender et al., 2008). In the mitochondria, PbPrx1 might play a role in protecting against ROS that is generated as a result of protein misfolding and aggregate formation (Weids and Grant, 2014).

To gain insights into the physiological roles of PbPrx1, its gene expression pattern was characterized when yeast cultures were exposed to organic and inorganic hydroperoxides. Our data showed a consistent time-dependent induction in the Pb*PRX1* gene expression when *P. brasiliensis* yeasts were exposed to the organic hydroperoxides *t*-BOOH and CHP, with peaks by 15 min of exposure of 8.5-fold and 5.45-fold increase, respectively. For *t*-BOOH, for instance, this value was 2–4-fold the induction caused by H_2_O_2_ (2-fold), which is in agreement with the fact that PbPrx1 showed higher affinity for organic hydroperoxides. Similarly, the *C. albicans* Prx1 (CaPrx1) peroxidase activity is able to reduce both *t*-BOOH and H_2_O_2_, but intracellular reactive oxygen species accumulate only when *prx1*Δ is treated with *t*-BOOH, indicating that its cellular function is more specific to organic hydroperoxides (Srinivasa et al., 2012). The increase in the expression of 1-Cys Prx1 upon oxidative stress was also described in *A. fumigatus*. The three 1-Cys Prx genes *PRX1, PRXB, and PRXC*, which show high activity against H_2_O_2_, were induced in a time-dependent manner when Paraquat was used as the oxidant molecule (Rocha et al., 2018).

The importance to study pathogen enzymes with antioxidant properties comes from the fact that tolerance to oxidative stress is an important trait of virulence in several microorganisms (Banin et al., 2003; Piacenza et al., 2013; Kaihami et al., 2014; Rocha et al., 2018). For that reason, they could also be targets for antifungal agents. In *C. albicans*, the Prx1 ortholog (Tsa1) is found in the cell wall specifically in the pathogenic hyphal phase, while the protein localizes in the cytosol and nucleus of yeast cells, pointing to a role in pathogenicity (Urban et al., 2003). In *P. brasiliensis*, attenuated yeast cells recovered their virulence after serial passages in mice and this process positively modulated the fungal antioxidant repertoire (Castilho et al., 2018). Additionally, proteins involved in the oxidative stress response in *P. brasiliensis* yeast cells were up-regulated during macrophage infection (Parente-Rocha et al., 2015).

Together, our results reveal that PbPrx1 is a peroxidase widely distributed inside (cytosol and mitochondria) and outside (cell wall and extracellular vesicles) *P. brasiliensis* cells. It is highly reactive with organic hydroperoxides (OHPs) and strongly induced by these oxidants, thus suggesting a role of importance in protecting *P. brasiliensis* against insults caused by organic hydroperoxides.

## Data Availability Statement

All datasets presented in this study are included in the article/Supplementary Material.

## Ethics Statement

The animal study was reviewed and approved by the Ethics Committtee for Research (UNIFESP) numbers: CEP 0366/07 and CEP 6379211014.

## Author Contributions

LL, GG, NL, and CO performed the cloning, antibody production, localization, and qRT-PCR experiments. CB, GN, and MT performed the biochemical characterization experiments. LL, CB, MO, and RP drafted the manuscript. All authors critically reviewed and approved the final manuscript.

## Conflict of Interest

The authors declare that the research was conducted in the absence of any commercial or financial relationships that could be construed as a potential conflict of interest.

## Supplementary Material

Supplementary Figure 1 Recombinant protein purification by IMAC. **(A)** rPbPrx1 expression was performed using *E. coli* Tuner (DE3) containing the pET28PP-*pbPRX1* vector. The protein purification was performed using IMAC: lanes 2–6 represent imidazol gradient progressive elution fractions; lane 6 shows the purified protein used in the experiments. The expressed protein in pHIS1–*pbPRX1* was purified in a Ni-NTA column and the profile of the purified protein was similar to that in lane 6. **(B)** *S. cerevisiae* Trx1 was expressed in *E. coli* BL 21 (DE3), purified by the boiling method and size exclusion chromatography using a HiLoad 16/600 Superdex 75 (GE Healthcare, Piscataway, USA). The elution fractions are shown; fractions 11-14 were pooled for use in the experiments. **(C)** *S. cerevisiae* Tsa1 was expressed in *E. coli* BL 21 (DE3) e purified by IMAC HiPrep IMAC FF Column (GE Healthcare). The elution fractions are shown; fractions 5-8 were pooled for use in the experiments. **(D)** Baker yeast TrxR1 was expressed in *E. coli* BL 21 (DE3) e purified by IMAC HiPrep IMAC FF Column (GE Healthcare). The elution fractions are shown; fractions 6–8 were pooled for use in the experiments. Lane 1 of each gel: unstained Protein MW Marker (Thermo Fisher Scientific).

Supplementary Figure 2 Amino acid alignment of 1-Cys Prx1 deduced sequences from pathogenic fungi and other species reveal conserved structural elements with *P. brasiliensis* Prx1. Amino acid sequence alignment was performed using ClustalΩ and graphic representation was generated using Jalview. Identical residues are shaded in blue and sequence similarity is indicated by a blue gradient based on the physicochemical characteristic conservation of the amino acid. The sequences correspond to the following species: Prx1_Pb = *P. brasiliensis* (NCBI accession number: XP_010758730.1); Prx1_Pl = *P. lutzii* (XP_002794671.1); Prx1_Hc = *Histoplasma capsulatum* (EEH07081.1); Prx1_Bd = *Blastomyces dermatitidis* (EEQ85711.1); Prx1_An = *Aspergillus niger* (XP_001401704.2); Prx1_Ao = *A. oryzae* (XP_001821217.1); Px1_Af = *A. fumigatus* (XP_747511.1); Prx1_Ca = *Candida albicans* (XP_717002.1); Prx1_Ct = *C. tropicalis* (XP_002550813.1); Prx1_Sc = *Saccharomyces cerevisiae* (NP_009489.1); Prx1_Pp = *Picchia pastoris* (XP_002490091); Prdx6_Hs = *Homo sapiens* (P30041.3); Prdx6_Rn = *Rattus novergicus* (O35244.3); Pdrx6_Mm = *Mus musculus* (O08709.3). The red line denotes the *N*-terminal mitochondrial signal peptide. The green box highlights the phospholipase A2 (PLA2) motif and the green asterisk the catalytic Ser residue. The red box denotes the conserved P-V/I-C-T-T/S-E signature (catalytic cysteine marked by a red asterisk) of the 1-Cys Prx1 sequences.

Supplementary Figure 3 PLA2 activity evaluation using NOBA. The assay was performed at 25°C in reaction mixtures containing 20 mM Tris, pH 7.8, 0.001 mg rPbPrx1, 150 mM NaCl, 10 mM CaCl, 0.1 mg/ml 3-(octanoyloxy) benzoic acid (NOBA). The absorbance was monitored at 425 nm for 60 min.

Supplementary Table 1 PbPrx1 thermal shift analysis by circular dichroism.
