# Supplementary figures and images for: The Human Pathogen Paracoccidioides brasiliensis Has a Unique 1-Cys Peroxiredoxin That Localizes Both Intracellularly and at the Cell Surface
Source: Front Cell Infect Microbiol. 2020 Aug 4;10:394. doi: 10.3389/fcimb.2020.00394 (PMC7417364; doi:10.3389/fcimb.2020.00394)

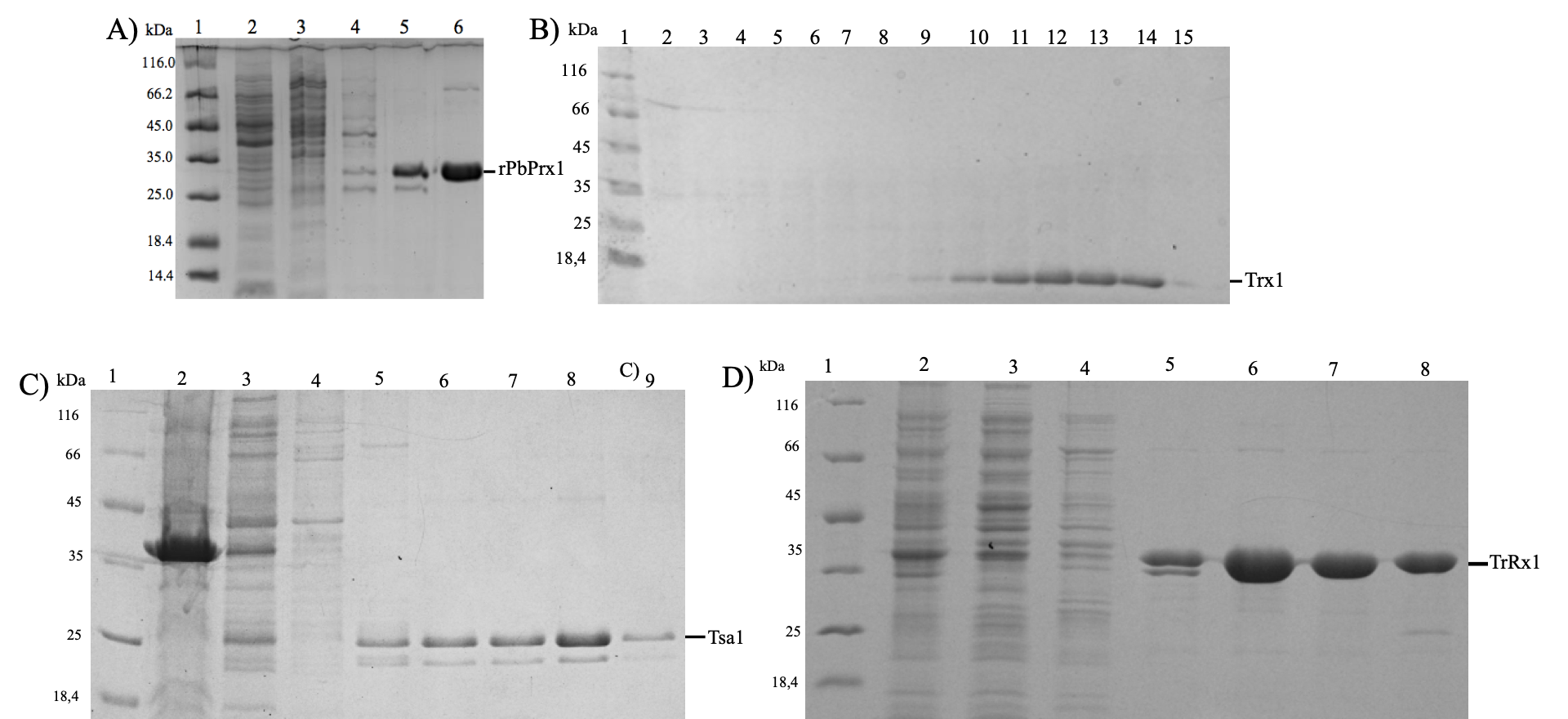

Supplement: Supplementary Figure 1 — Recombinant protein purification by IMAC. (A) rPbPrx1 expression was performed using E. coli Tuner (DE3) containing the pET28PP-pbPRX1 vector. The protein purification was performed using IMAC: lanes 2–6 represent imidazol gradient progressive elution fractions; lane 6 shows the purified protein used in the experiments. The expressed protein in pHIS1–pbPRX1 was purified in a Ni-NTA column and the profile of the purified protein was similar to that in lane 6. (B) S. cerevisiae Trx1 was expressed in E. coli BL 21 (DE3), purified by the boiling method and size exclusion chromatography using a HiLoad 16/600 Superdex 75 (GE Healthcare, Piscataway, USA). The elution fractions are shown; fractions 11-14 were pooled for use in the experiments. (C) S. cerevisiae Tsa1 was expressed in E. coli BL 21 (DE3) e purified by IMAC HiPrep IMAC FF Column (GE Healthcare). The elution fractions are shown; fractions 5-8 were pooled for use in the experiments. (D) Baker yeast TrxR1 was expressed in E. coli BL 21 (DE3) e purified by IMAC HiPrep IMAC FF Column (GE Healthcare). The elution fractions are shown; fractions 6–8 were pooled for use in the experiments. Lane 1 of each gel: unstained Protein MW Marker (Thermo Fisher Scientific). [file Image_1.PNG]

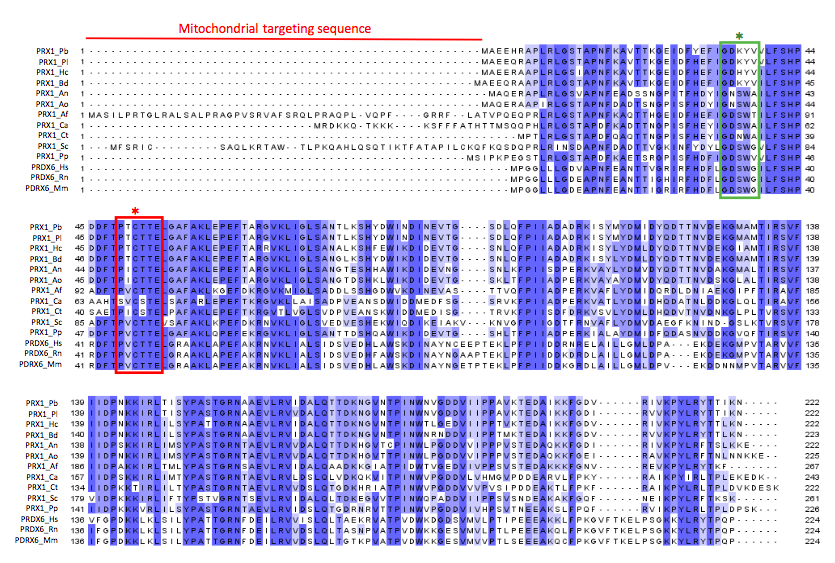

Supplement: Supplementary Figure 2 — Amino acid alignment of 1-Cys Prx1 deduced sequences from pathogenic fungi and other species reveal conserved structural elements with P. brasiliensis Prx1. Amino acid sequence alignment was performed using ClustalΩ and graphic representation was generated using Jalview. Identical residues are shaded in blue and sequence similarity is indicated by a blue gradient based on the physicochemical characteristic conservation of the amino acid. The sequences correspond to the following species: Prx1_Pb = P. brasiliensis (NCBI accession number: XP_010758730.1); Prx1_Pl = P. lutzii (XP_002794671.1); Prx1_Hc = Histoplasma capsulatum (EEH07081.1); Prx1_Bd = Blastomyces dermatitidis (EEQ85711.1); Prx1_An = Aspergillus niger (XP_001401704.2); Prx1_Ao = A. oryzae (XP_001821217.1); Px1_Af = A. fumigatus (XP_747511.1); Prx1_Ca = Candida albicans (XP_717002.1); Prx1_Ct = C. tropicalis (XP_002550813.1); Prx1_Sc = Saccharomyces cerevisiae (NP_009489.1); Prx1_Pp = Picchia pastoris (XP_002490091); Prdx6_Hs = Homo sapiens (P30041.3); Prdx6_Rn = Rattus novergicus (O35244.3); Pdrx6_Mm = Mus musculus (O08709.3). The red line denotes the N-terminal mitochondrial signal peptide. The green box highlights the phospholipase A2 (PLA2) motif and the green asterisk the catalytic Ser residue. The red box denotes the conserved P-V/I-C-T-T/S-E signature (catalytic cysteine marked by a red asterisk) of the 1-Cys Prx1 sequences. [file Image_2.TIFF]

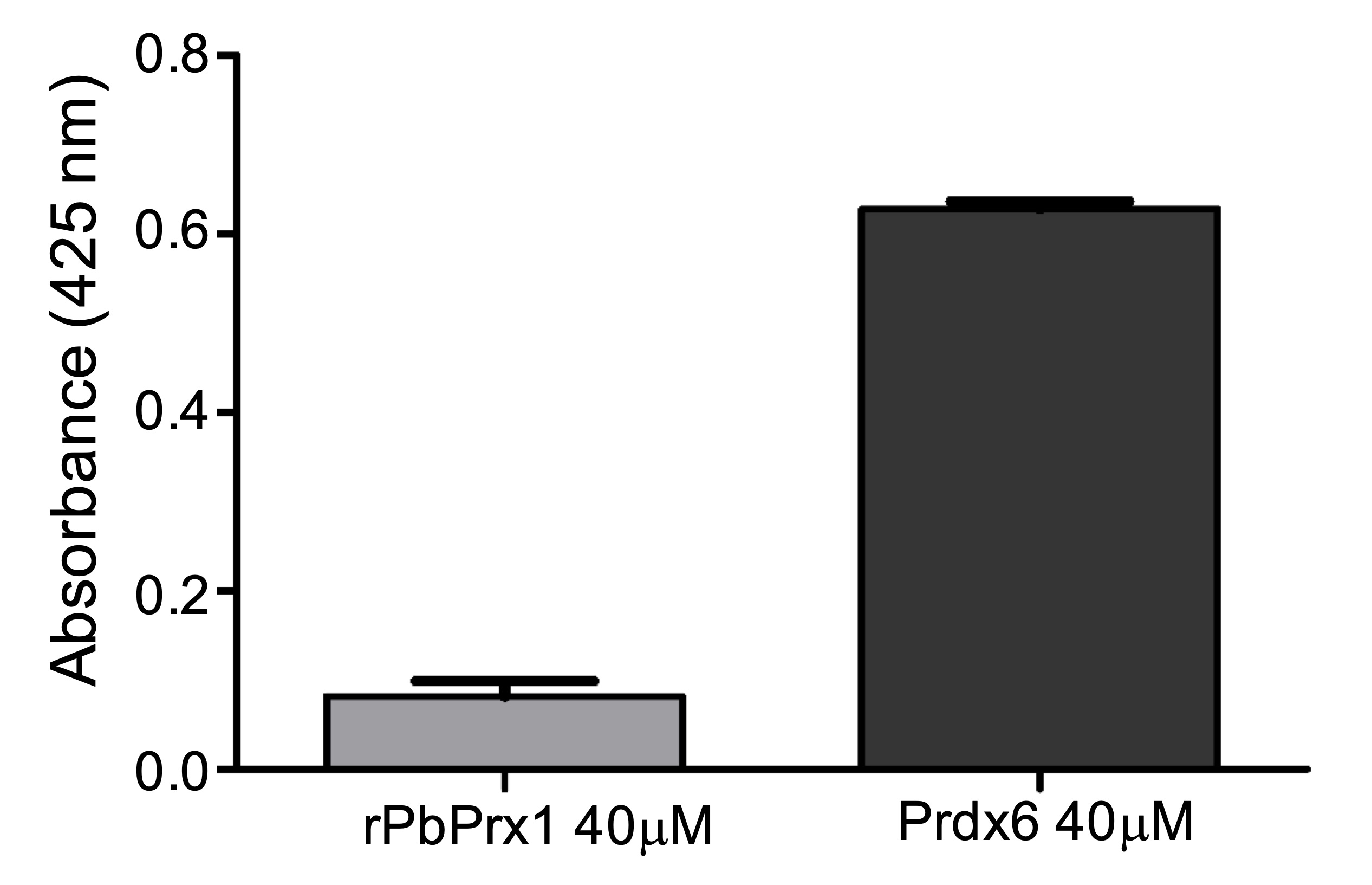

Supplement: Supplementary Figure 3 — PLA2 activity evaluation using NOBA. The assay was performed at 25°C in reaction mixtures containing 20 mM Tris, pH 7.8, 0.001 mg rPbPrx1, 150 mM NaCl, 10 mM CaCl, 0.1 mg/ml 3-(octanoyloxy) benzoic acid (NOBA). The absorbance was monitored at 425 nm for 60 min. [file Image_3.jpg]
