# Supplementary material for: The Human Pathogen Paracoccidioides brasiliensis Has a Unique 1-Cys Peroxiredoxin That Localizes Both Intracellularly and at the Cell Surface
Source: Front Cell Infect Microbiol. 2020 Aug 4;10:394. doi: 10.3389/fcimb.2020.00394 (PMC7417364; doi:10.3389/fcimb.2020.00394)
Supplement: Supplementary Table 1 — PbPrx1 thermal shift analysis by circular dichroism. [file Table_1.DOCX]

**Supplementary Table 1.** PbPrx1 thermal shift analysis by circular dichroism

|  | **20 °C** | **30 °C** | **40 °C** | **50 °C** | **60 °C** | **70 °C** | **80 °C** |
| --- | --- | --- | --- | --- | --- | --- | --- |
| α-helix (%) | 36.2 | 35.8 | 29.5 | 31,3 | 22.1 | 19.9 | 19.5 |
| β-strand (%) | 12.2 | 12.4 | 18 | 17,00 | 31 | 35.4 | 36.4 |
| β-turn (%) | 14.6 | 14.7 | 16.3 | 16.4 | 17.5 | 17.4 | 17.4 |
| Random coil (%) | 36.9 | 37.1 | 36.2 | 35.3 | 29.4 | 27.3 | 26.8 |
